# Why Does the “Sinner” Act Prosocially? The Mediating Role of Guilt and the Moderating Role of Moral Identity in Motivating Moral Cleansing

**DOI:** 10.3389/fpsyg.2016.01317

**Published:** 2016-09-08

**Authors:** Wan Ding, Ruibo Xie, Binghai Sun, Weijian Li, Duo Wang, Rui Zhen

**Affiliations:** ^1^School of Psychology, Beijing Normal UniversityBeijing, China; ^2^College of Teacher Education, Zhejiang Normal UniversityJinhua, China; ^3^School of Social Science, Policy and Evaluation, Claremont Graduate University, ClaremontCA, USA

**Keywords:** moral cleansing, moral compensation, ethical dissonance, moral identity, guilt, moral self-image

## Abstract

Numerous studies have found that people tend to commit prosocial acts subsequent to previous immoral acts, as a response to the latter. This phenomenon is called moral cleansing or moral compensation. However, the specific mechanism how previous immoral acts motivate moral compensatory behaviors is still not fully understood. This study aimed to examine the roles of guilt and moral identity in the relation between previous immoral acts and subsequent prosocial behaviors to clarify the mechanism. Based on the extant research, the current study proposed a moderated mediation model to illustrate the process of moral cleansing. Specifically, a previous immoral act motivates guilt, which further leads to subsequent prosocial behaviors, while moral identity facilitates this process. The participants were primed by a recalling task (immoral act vs. a neutral event). The results support the hypothesized model and provide a framework that explains moral cleansing by integrating the roles of guilt and moral identity. These findings highlight the dynamic nature of people’s morality with regard to how people adapt moral behaviors to protect their moral self-image.

## Introduction

Past research shows us that people behave dishonestly, but at the same time manage to perceive themselves as good and honest (Mazar et al., 2008; Jordan et al., 2011; Shalvi et al., 2015). According to the common theoretical model of self-maintenance, people are torn between their wish to be moral and the temptation to profit from dishonesty. This conflict is termed ethical dissonance (Barkan et al., 2012, 2015). Ethical dissonance elicits intense psychological tension and poses a threat to people’s self-concept and well-being (Mulder and Aquino, 2013; Barkan et al., 2015). To reduce the tension and maintain the moral self, people use justification mechanisms. Some justifications can take place before people commit the ethical violation, such as ambiguity of rules, the prosocial nature of the act, and moral licensing (Shalvi et al., 2015). They enable people to excuse misbehaviors as less immoral and thus reduce anticipated ethical dissonance. More often, other justifications emerge after people’s moral misconduct, in order to minimize the experienced dissonance, wipe out feelings of guilt, and cleanse the self.

Moral cleansing, also known as moral compensation, is a set of compensating moral or worthy actions that cancels out the ethical violation that preceded it, allowing a person to turn a new leaf (Zhong and Liljenquist, 2006; Sachdeva et al., 2009). This effect has been largely documented in previous research and shows the inconsistency in morality (Jordan et al., 2011; Conway and Peetz, 2012; Gino et al., 2015). For instance, it was showed that people who recollected their past immoral behaviors showed high enthusiasm for prosocial activities and less dishonesty than those who recalled their past moral behaviors (Jordan et al., 2011; Gino et al., 2015). Conway and Peetz (2012) also found that participants who were asked to write their own past immoral behaviors reported stronger willingness to offer help and donate more money to charity. Since moral cleansing has been demonstrated by various studies, it is important to explore why “sinners” act prosocially after their misdeeds. In this context, we need to consider two key factors.

## Guilt and Moral Cleansing

Guilt is one of the negative consequences of ethical dissonance (Tangney, 1990; Zhong and Liljenquist, 2006). It is a measurable aspect of the psychological tension of ethical dissonance (Tangney et al., 2007). Research has shown that guilt has a unique role in ethical dissonance (Gino et al., 2009; Sheikh and Janoff-Bulman, 2010; Xu et al., 2014). As the dissonance is more acute, the feelings of guilt increase, which may put more pressure on the individual to reduce the threat of ethical dissonance on the self. Cleansing can be an effective way to reduce that tension (Barkan et al., 2015). Thus, as people feel more guilt, their tendency for cleansing will increase. Specifically, we hypothesize that an immoral act motivates guilt, which further leads to subsequent prosocial behaviors.

## Moral Identity and Moral Cleansing

Interestingly, people can feel more or less guilt. Some people may feel very guilty because they took a newspaper without paying for it, while other people may do worse things like stealing thousands and feel less guilty. This indicates the individual differences in people’s response to their past immoral act, consistently immoral act (e.g., stealing), or inconsistently compensatory act (e.g., donating money or helping others; Zhong and Liljenquist, 2006; Martens et al., 2010). Past research has shown that everyone wants to be moral and sees morality as an important part of their identity (Blasi, 1993). Fine-tuning this concept, Aquino and Reed (2002) referred to the centrality of moral identity to one’s self-concept. They showed that, for some people, moral identity is an important and central part of their general identity, whereas for other people (who also see themselves as moral) this component is less central (Aquino and Reed, 2002; Aquino et al., 2009). We hypothesize that, as moral identity is more central to one’s self-concept, he or she will be more susceptible to ethical dissonance, experience more psychological tension, and act more prosocially to minimize this distress.

Moral identity is defined as “a self-conception organized around a set of moral traits” and it reflects the self-importance of morality (Aquino and Reed, 2002). The trait-based definition stems from Blasi’s (1984) thought that some moral traits (e.g., caring or helpful) may stand more centrally in one’s self-concept than others (e.g., honest or generous). Aroused by the drive to maintain consistency between self-conception and action, moral identity contributes to motivate moral actions, as a self-regulation (Blasi, 2004; Aquino et al., 2009; Brooks et al., 2013). Specifically, people spontaneously compare their current actions with their moral self-conception, and once the comparative deficit or the threat to their moral self is detected, the psychological distress is generated (Barkan et al., 2015; West and Zhong, 2015). This suggests that the centrality of moral identity facilitates the evoking of guilt and compensatory behavior in ethical dissonance (Zhong et al., 2010). Further, Mulder and Aquino (2013) conducted an empirical research on the relationship between moral identity and moral cleansing. The results showed a facilitating effect of moral identity on moral compensatory behavior.

## The Current Study

Based on the theoretical models and existing findings related to ethical dissonance, we presumed the process of moral cleansing by integrating the roles of guilt and moral identity simultaneously. Specifically, when committing an immoral or indecent act, people will experience stronger discrepancy between their behaviors and existing moral identity, which can elicit guilt (Jordan et al., 2011; Mulder and Aquino, 2013). The higher a person’s moral identity is, the stronger guilt he/her feels. The desire to reduce the guilt can motivate them to engage in moral actions to protect their moral self-image, or in other words, to wash away their sins (West and Zhong, 2015).

Correspondingly, we proposed a moderated mediation model to illustrate the roles of guilt and moral identity in moral cleansing. To be specific, guilt mediates the relationship between previous immorality and moral compensatory acts, whereas moral identity plays a moderating role in this process. It is necessary to examine moral cleansing from a cross-cultural perspective before illustrating its process, and it has never been demonstrated among Chinese who grew up in an oriental cultural background. Thus, in this study we examined this assumption in a sample of Chinese young adults. Above all, this study aimed (1) to examine the moral cleansing effect in a Chinese sample, and (2) to clarify the roles of guilt and moral identity and their interplay in moral cleansing. The corresponding hypotheses were as follows:

*Hypothesis 1*: Previous immorality will motivate the tendency to offer help.
*Hypothesis 2*: Guilt will mediate the relationship between previous immorality and moral compensatory acts, and moral identity will play a positive moderating role in this process.

## Materials and Methods

### Participants

In total, 360 Chinese adults participated in this online study via Sojump. They were provided a chance to win a raﬄe prize of ¥100 (about $15). On an average, these participants were aged 23.74 years (*SD* = 5.98 years), ranging from 18 to 38 years. Further, 169 participants (47%) were male and 210 participants (58%) were undergraduate students.

### Design

To examine the moral cleansing effect, the participants were randomly distributed to different recalling tasks: recalling their own previous immoral acts for the primed group (*n* = 180) and recalling their own neutral acts for the unprimed group (*n* = 180). To further clarify the association among previous immoral behavior, guilt, moral identity, and moral compensatory behavior, the last three variables were measured and the immorality of previous immoral behavior was evaluated on a 4-point scale (0 = neutral, 1 = a little immoral, 2 = moderately immoral, and 3 = very immoral).

### Measures

#### Moral Identity

The internalization subscale of the moral identity measure (Aquino and Reed, 2002) was used to assess the centrality of moral identity. This subscale of the two-dimensional instrument has been shown to tap into the degree to which moral traits are central to the self-concept (Aquino and Reed, 2002) and has been used in several studies on moral functioning (Aquino et al., 2009; Mulder and Aquino, 2013). The measure presents participants with a list of nine adjectives that might describe a person (generous, helpful, hardworking, honest, kind caring, compassionate, fair, and friendly) and then asks them to “visualize the kind of person who has these characteristics and imagine how that person would think, feel, and act.” After being asked to think about someone who possesses these traits, participants were presented with the five items. Sample items included, “Being someone who has these characteristics is an important part of who I am,” and “It would make me feel good to be a person who has these characteristics.” Each of the items was answered on a 7-point Likert-type scale (1 = strongly disagree and 7 = strongly agree). Then, the five items were averaged to determine the moral identity score for each participant (α = 0.85).

#### Priming Manipulation Using the Recalling Task

The priming manipulation used a procedure designed by Zhong and Liljenquist (2006), which had been used in several studies (Barkan et al., 2012; Mulder and Aquino, 2013; Jordan et al., 2015). At the beginning of the priming, all participants read instructions stating that the researchers were interested in exploring people’s memory of daily life events. Then, the participants in the primed group was asked to recall one of their past unethical events and to describe any details, feelings, or emotions they experienced, while participants in the control group were asked to write down certain occurrences that had happened since a week ago until the present (Mulder and Aquino, 2013). We coded the immorality of the recalled acts by the method adapted from Jordan et al. (2011). According to Kaptein’s (2008) definition, for immoral behavior: “violating significant (social) moral norms that are acceptable to the larger community,” the immorality of the recalled acts was evaluated on a 4-point scale (0 = neutral, 1 = a little immoral, 2 = moderately immoral, 3 = very immoral). This method helped in understanding the association between previous immorality and compensatory behavior. The intraclass correlation coefficient (ICC = 0.84) showed a high initial interrater reliability; three coders discussed discrepancies to arrive at a consensus.

#### Guilt

At the end of the recall task, all participants were presented with the guilt scale (GS; Ding, 2015) to measure their current feelings of guilt, which was adapted from Tangney et al. (1996) and Lewis’s (1971) measurements. The guilt scale consists of 16 items, with five items in the dimensions of realizing one’s own error (α = 0.86), six items in the dimension of feeling (α = 0.91), and five items in the dimensions of behavior tendency (α = 0.83). Respondents answered each question on a 7-point Likert scale (1 = strongly disagree and 7 = strongly agree). The Cronbach’s alpha reliabilities indicated that the GS achieved optimal levels as per psychometric requirements.

#### The Tendency of Volunteering Behavior

A method revised from Schnall et al.’s (2010) measure was utilized to measure the tendency of volunteering behavior. Specifically, after completing the guilt scale, the participants were informed that the study had ended. Then, a window popped up to show the “Ask for help” situation. The window stated, “There is another survey for which we need your help, without any pay. Any amount of help would be greatly appreciated. You are free to decide whether you will be willing to help us and to choose the time you wish to spend on the survey before the survey starts.” The time ranged from 0 to 120 min, at intervals of 10 min. According to Korsgaard et al.’s (2010) study, the experimenter should state that the participants (a) would receive no incentive for participating and (b) were not obligated to participate. As Korsgaard et al. (2010) explain, it is a valid measure to assess the participants’ volunteering behavior. The given answers were encoded to 0–12 (ranging from *volunteering no time* to *2 h*, in 10-min increments), according to Oswald’s (1996, 2002) method. Thus, the tendency toward volunteering behavior was measured and encoded.

### Procedure

The Institutional Review Board of Zhejiang Normal University in China approved the protocol of the present study, including the consent procedure. We also obtained consent from our participants. All materials and measures were completed online, anonymously. At the beginning of the on-line survey, the moral identity of all participants was measured, after which, some filler questionnaires (about 30 items) unrelated to morality were filled. Then, the participants in the two groups were primed or controlled with different recalling tasks, respectively. Next, the guilt scale was used to measure the guilt of all the participants. Last, all subjects participated in a test for participants’ prosocial intentions in a simulated “Ask for help” situation. In total, nine participants were excluded for failing to recall their previous immoral acts in the primed group, and 13 participants were excluded for their invalid questionnaires (six in the primed group and seven in the unprimed group), which involved choosing the same, completely random, or contradicting options for the items.

## Results

### Preliminary Analyses

Before testing our predictions, we described the study variables using means and standard deviations of the measures, which have been shown in **Table 1**. Then, the Pearson correlation analysis was conducted to explore the basic relationships between previous immorality, guilt, moral identity, and helping time. These results are also presented in **Table 1**, indicating that the compensatory prosocial behavior was positively correlated with previous immorality, guilt, and moral identity.

**Table 1:** Descriptive statistics and correlation matrix.

	Content of recalling task								
	Neutral (*n* = 173)						Immoral (*n* = 165)					
	*M*	*SD*	1	2	3	4	*M*	*SD*	1	2	3	4
1. Immorality	0	0	-				1.97	0.82	-			
2. Guilt	0.60	0.14	-	-			5.20	1.27	0.55^∗∗^	-		
3. Moral identity	4.97	0.86	-	0.48^∗∗^	-		5.04	0.89	0.08	0.52^∗∗^	-	
4. Helping time	3.89	1.81	-	0.33^∗∗^	0.19^∗^	-	2.80	1.64	0.27^∗∗^	0.30^∗∗^	0.23^∗∗^	-

*^∗^*p* < 0.05, ^∗∗^*p* < 0.01.*

*Morality of the recalled act ranges from 0 (neutral) to 3 (very immoral). Guilt scores range from 1 (strongly disagree e) to 7 (strongly agree). Moral identity scores range from 1 (completely disagree) to 7 (completely agree). Helping time ranges from 0 (no help) to 12 (120 min).*

### Hypothesis Testing

The chi-square test and one-way ANOVA were conducted to examine Hypothesis 1. First, the chi-square test showed that 66.86% of the participants in the primed group offered help, whereas only 34.27% of participants in the unprimed group did so (χ*^2^* = 14.992, *p* < 0.001, Φ = 0.21). Then, the results of the ANOVA showed a significantly different tendency for engaging in volunteering behavior between the primed and control groups (primed group: 3.89 ± 1.81; control group: 2.80 ± 1.64; *t* = 5.71, *p* < 0.001, Cohen’s *d* = 0.63). As predicted, relative to the control group, recalling previous immoral behavior in the primed group motivated moral compensatory intentions. Before examining the hypothesized moderated mediation model, we tested the effect of previous immorality on subsequent prosocial behavior using a regression analysis. The result (*B* = 0.25, *SE* = 0.04, *p* < 0.001) indicated that every 1-unit increase in the previous immorality predicted a 0.25-unit increase in moral compensatory behavior.

**FIGURE 1:**
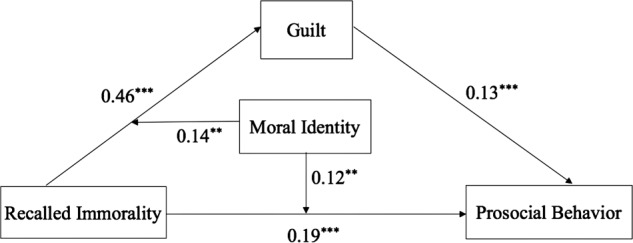
**The moderated mediation model.** ^∗∗^*p* < 0.01, ^∗∗∗^*p* < 0.001.

To examine the association among previous immorality, guilt, moral identity, and compensatory behavior, we tested the moderated mediation model (Hypothesis 2) according to Muller et al.’s (2005) multiple regression analysis process with centered variables (Aiken et al., 1991). The regression analysis was conducted using the enter method. Bootstrap confidence intervals (CI) were computed for the regressions coefficients and 95% CI not containing 0 indicates significant results (Erceg-Hurn and Mirosevich, 2008). The results have been presented in **Table 2** and **Figure 1**.

**Table 2:** The regression results for moderated mediation model.

Predictor variables	The first step Helping time			The second step Guilt			The third step Helping time		
	*B*	*SE*	95%CI	*B*	*SE*	95%CI	*B*	*SE*	95%CI
X: Previous immorality (PI)	0.25	0.04	[0.16, 0.34]	0.46	0.03	[0.39, 0.53]	0.19	0.03	[0.12, 0.26]
Mo: Moral identity (MI)	0.08	0.04	[-0.02, 0.18]	0.09	0.03	[-0.01, 0.19]	0.07	0.04	[-0.01, 0.13]
XMo: PIMI	0.11	0.04	[0.03, 0.19]	0.14	0.03	[0.08, 0.20]	0.12	0.04	[0.04, 0.22]
Me: Guilt (G)	-	-		-	-		0.13	0.03	[0.05, 0.21]
MeMo: GMI	-	-		-	-		-0.05	0.03	[-0.10, 0.01]

Adj *R*^2^	0.22			0.48			0.35		
*F*	19.21			144.18			23.47		

*Mo, moderator variable; Me, mediator variable.*

First, previous immorality had a significantly indirect effect on subsequent prosocial behavior through guilt. To be specific, the path from previous immorality to guilt and the path from guilt to subsequent prosocial behavior were significant. The indirect effect (from previous immorality to the subsequent prosocial behavior through guilt) equaled 0.06, which was the product of 0.46 (the path from previous immorality to guilt) and 0.13 (the path from guilt to prosocial behavior). In addition, the direct effect of previous immorality on prosocial behavior reduced from 0.25 to 0.19 after guilt was added to the model. This indicated the partial mediating effect of guilt in the relationship between previous immorality and prosocial behavior, and the mediating effect made up 24% (0.06/0.25) of the total effect. Moreover, for the analysis on helping time, the adjusted *R*^2^ increased from 0.22 to 0.35 when guilt was added in the third step. That is, guilt had an additional *R*^2^ value of 13%.

**FIGURE 2:**
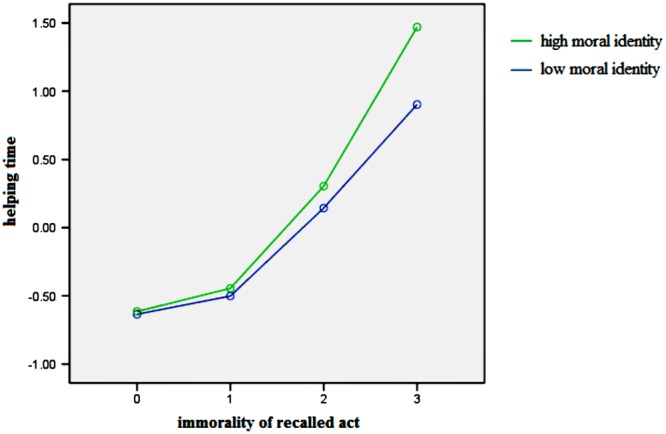
**The relationship between recalled immorality and helping time for high and low levels of moral identity**.

**FIGURE 3:**
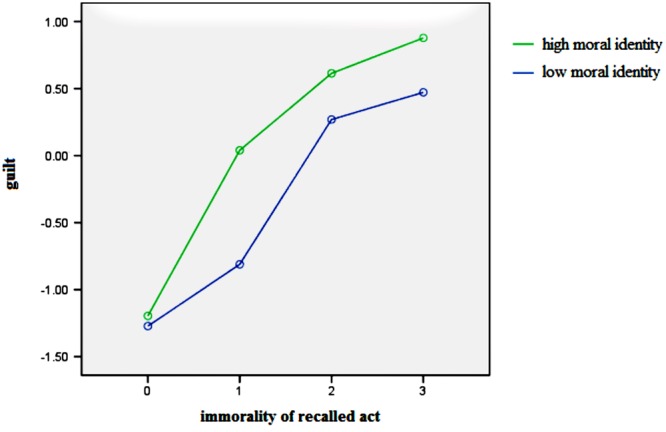
**The relationship between recalled immorality and guilt for high and low levels of moral identity**.

Secondly, the moderating effect of moral identity was shown on the direct and indirect path of moral compensation. Specifically, the interaction of previous immorality and moral identity (PIMI) had a significant effect on guilt and prosocial behavior. To present the moderating role of moral identity in moral compensation, we plotted the two interactions in **Figures 2** and **3**, at different levels of previous immorality (0–3) and moral identity centrality (1 *SD* above and below the mean for high and low levels). **Figure 2** illustrates the effects of previous immorality on subsequent helping time while **Figure 3** shows the effects of previous immorality on guilt for high and low levels of moral identity centrality. As shown in **Figure 2**, there is a stronger moral cleansing effect for people who have a high centrality of moral identity (high-MI) than for people who have a low centrality of moral identity (low-MI) at Level 3 [*M*_high-MI_ = 1.49, *M*_low-MI_ = 0.87, *t*_(39)_ = 1.97, *p* < 0.05, Cohen’s *d* = 0.62]. As presented in **Figure 3**, stronger guilt was elicited for high-MI people than for low-MI people at almost all levels of immorality [Level 1: *M*_high-MI_ = 0.06, *M*_low-MI_ = -0.83, *t*_(30)_ = 2.54, *p* < 0.01, Cohen’s *d* = 0.89; Level 2: *M*_high-MI_ = 0.64, *M*_low-MI_ = 0.24, *t*_(48)_ = 1.43, *p* < 0.10, Cohen’s *d* = 0.40; Level 3: *M*_high-MI_ = 0.94, *M*_low-MI_ = 0.43, *t*_(39)_ = 1.70, *p* < 0.05, Cohen’s *d* = 0.51].

## Discussion

This study had revealed a moral cleansing effect among Chinese young adults. More importantly, the results supported the assumptive model and provided a framework for explaining moral compensation by integrating the roles of guilt and moral identity. Specifically, previous immorality elicits the feeling of guilt, which further motivates moral compensatory behavior to alleviate this psychological distress. Moral identity facilitates the process of moral cleansing directly or through eliciting strong guilt.

Moral compensation exists both in eastern and western cultures, indicating that moral compensation is a cross-cultural phenomenon. Specifically, compared with the percentages of participants (66.7 and 33.3%) who chose a cleansing product (i.e., an antiseptic wipe, versus a non-cleansing product, i.e., a pencil) in Zhong and Liljenquist’s (2006) study, the present study revealed a similar percentage in the primed (66.86%) and unprimed (34.27%) groups in terms of the tendency to offer help. However, a difference was found between the percentage of those who offered help in the present study and of those who did so (73.9 and 40.9%) in Zhong and Liljenquist’s (2006) study. These comparisons showed a similar effect of moral compensation and a different level of helping behavior in participants from different cultural backgrounds. Extending previous findings (Zhong and Liljenquist, 2006; Conway and Peetz, 2012), the present study also found the quantitative association that a 1-unit increase in the previous immorality predicted a 0.25-unit increase in moral compensatory behavior. The findings indicated that the higher level of previous immorality that people recalled, the more prosocial behavior they would commit subsequently.

Previous immorality motivates moral compensatory behavior through guilt. This finding extended our existing understanding of the effect of guilt (Tangney et al., 2007; Gino et al., 2009; Sheikh and Janoff-Bulman, 2010) and supported the guilt-motivation perspective of moral cleansing (Zhong and Liljenquist, 2006; Xu et al., 2014). Specifically, the mediation model showed two sub-processes of moral cleansing. That is, previous immorality firstly leads to a sense of guilt, and secondly, this feeling of psychological distress motivates moral compensatory behavior. Further, the more immoral the recalled action is, the stronger is the feeling of guilt, and the higher is the prosocial behavior that is elicited. Besides, previous immorality also had a direct effect on moral compensatory behavior after controlling for the role of guilt. This suggested a possibility that previous immorality motivates moral cleansing directly as well as through other psychological tension.

The centrality of moral identity can facilitate the direct process from previous immorality to moral compensatory behavior. The findings supported Zhong et al.’s (2010) assumption and were consistent with the results of Mulder and Aquino’s (2013) research. Extending Mulder and Aquino’s (2013) study, the present study examined the moderating role of moral identity in the process of moral cleansing, with the role of guilt considered. The results showed that moral identity could act as a moderator in the direct process from previous immorality to moral compensatory behavior, as presented in **Figure 1**. That is, as compared with a person with low centrality of moral identity (low-MI), a high-MI person (moral identity is more central to him/her) is more inclined to compensate for their previous immorality and subsequently act more prosocially. Additionally, we presumed that the significantly direct process from previous immorality to moral compensatory behavior might be attributed to some other moral emotions (e.g., shame) or to psychological tension. Then, the centrality of moral identity may promote moral cleansing through evoking a feeling of shame or distress, which needs further exploration in future research.

More importantly, our finding on the interplay between guilt and moral identity helps to explain how guilt was elicited and influenced by moral identity in moral cleansing. It contributed to an adequate understanding of moral compensation. The results presented in **Table 2** and **Figure 3** indicated that the centrality of moral identity could facilitate the process from previous immorality to guilt. This finding supported the self-consistency theory (Barkan et al., 2015) and the self-comparison model of moral compensation (West and Zhong, 2015). When people recollect their own past immoral behavior, the inconsistency between one’s self-conception and real conduct will lead to a sense of incompleteness and guilt. That is to say, they will feel guilt when they find themselves falling short of their existing moral identity. Thus, when moral identity is more important/central, the discrepancy between moral self-perception and the immorality of the recalled event is more pronounced, and people experience higher levels of guilt.

Interestingly, the demonstrated moderated mediation also contributes to explain moral consistency, in addition to moral inconsistency (e.g., moral compensation). The findings of the current study can help to explain why immoral behavior was followed by immoral behavior for some people (Zhong and Liljenquist, 2006; Martens et al., 2010). For low-MI people, their immoral act is consistent with their low moral identity. Therefore, it is possible that low-MI people mostly have no or just a low extent of discrepancy between immoral acts and moral identity, so they will experience less guilt or psychological distress. Low-MI people often continue their immoral behavior, but not moral compensatory behavior, after immoral acts.

Above all, combined with previous findings (Mulder and Aquino, 2013; Xu et al., 2014) and the self-consistency theory, the present study proposed and tested the moderated mediation model to show the mechanism underlying moral cleansing. The findings clarified and highlighted the vital importance of moral identity and guilt in moral self-regulation and the equilibrium of moral behavior. Specifically, previous immorality could not only motivate moral compensatory acts directly, but also through guilt. Besides, moral identity could facilitate the processes of evoking guilt and subsequent prosocial behavior by previous immorality. To sum up, the present study revealed that moral cleansing was observed among Chinese participants, and the findings showed us a framework to explain moral compensation with reference to the interplay between guilt and moral identity.

Several limitations should be addressed here. First, the present study only focused on the role of guilt, which may ignore the effects of other moral emotions in the process of moral compensation, such as shame. Therefore, other mediators should be distinguished in future studies. Second, the tendency of subsequent prosocial behavior was used to indicate participants’ subsequent compensatory behavior. A gap between the tendency and actual behavior may affect the results. Hence, measures for the actual behavior should be considered in future studies. Third, the present study only focused on the internalization dimension of moral identity and did not put the role of the symbolization dimension (Jordan et al., 2011) into our consideration. Future research can integrate the two dimensions of moral identity to uncover the mechanism of moral cleansing.

Despite these limitations, to our knowledge, this is the first time to probe into the mechanism underlying moral compensation from a comprehensive perspective of combining guilt and moral identity. This study revealed a dynamic model on how people adapt moral behavior to protect their moral self-image. Furthermore, since the research was carried out on a Chinese population, it offers us a glimpse of the cross-cultural differences. Actually, it does not point at differences, but shows that despite the different cultural background, the same psychological processes of ethical dissonance and moral cleansing equally apply to Chinese participants. Finally, from the perspective of application, the present findings also have important implications for motivating the prosocial behaviors of “sinners.” Practical efforts should concentrate on eliciting the discrepancy between sinners’ desired state (moral identity) and their current state (recalling his own previous immorality) to induce their subsequent prosocial behaviors.

## Author Contributions

Conceived and designed the experiments: WD, BS, RX, and WL. Performed the experiments: WD and RX. Analyzed the data: WD. Contributed to the writing of the manuscript: WD, RX, DW, and RZ.

## Conflict of Interest Statement

The authors declare that the research was conducted in the absence of any commercial or financial relationships that could be construed as a potential conflict of interest.
